# Resveratrol Suppresses Constitutive Activation of AKT via Generation of ROS and Induces Apoptosis in Diffuse Large B Cell Lymphoma Cell Lines

**DOI:** 10.1371/journal.pone.0024703

**Published:** 2011-09-12

**Authors:** Azhar R. Hussain, Shahab Uddin, Rong Bu, Omar S. Khan, Saeeda O. Ahmed, Maqbool Ahmed, Khawla S. Al-Kuraya

**Affiliations:** Human Cancer Genomic Research, King Faisal Specialist Hospital and Research Center, Riyadh, Saudi Arabia; Chinese University of Hong Kong, Hong Kong

## Abstract

**Background:**

We have recently shown that deregulation PI3-kinase/AKT survival pathway plays an important role in pathogenesis of diffuse large B cell lymphoma (DLBCL). In an attempt to identify newer therapeutic agents, we investigated the role of Resveratrol (trans-3,4′, 5-trihydroxystilbene), a naturally occurring polyphenolic compound on a panel of diffuse large B-cell lymphoma (DLBCL) cells in causing inhibition of cell viability and inducing apoptosis.

**Methodology/Principal Findings:**

We investigated the action of Resveratrol on DLBCL cells and found that Resveratrol inhibited cell viability and induced apoptosis by inhibition of constitutively activated AKT and its downstream targets via generation of reactive oxygen species (ROS). Simultaneously, Resveratrol treatment of DLBCL cell lines also caused ROS dependent upregulation of DR5; and interestingly, co-treatment of DLBCL with sub-toxic doses of TRAIL and Resveratrol synergistically induced apoptosis via utilizing DR5, on the other hand, gene silencing of DR5 abolished this effect.

**Conclusion/Significance:**

Altogether, these data suggest that Resveratrol acts as a suppressor of AKT/PKB pathway leading to apoptosis via generation of ROS and at the same time primes DLBCL cells via up-regulation of DR5 to TRAIL-mediated apoptosis. These data raise the possibility that Resveratrol may have a future therapeutic role in DLBCL and possibly other malignancies with constitutive activation of the AKT/PKB pathway.

## Introduction

Diffuse Large B-cell Lymphoma (DLBCL) is the most common lymphoid malignancy and constitutes approximately 40% of all cases [1]. Despite improvement in treatment protocols, treatment has been shown to cure 50% of all cases and a large number of DLBCL cases remain refractory to treatment [2], [3]. Dysregulated survival pathways have been shown to contribute to aggressiveness of DLBCL. We have previously shown that PI3′-kinase/AKT signaling plays a pivotal role in pathogenesis of DLBCL and other cancer cells by activating AKT and it's down stream targets, FOXO-1, GSK-3 and Bad [4], [5], [6]. AKT prevents apoptosis by generating anti-apoptotic signals through modulation of the activity of various survival and pro-apoptotic molecules [7], [8].

The Redox system is an essential tool to maintain a balance between generation and elimination of Reactive oxygen species (ROS) under physiological conditions in cells [9]. ROS refers to oxygen-containing breakdown products of molecular oxygen that are highly reactive and are able to damage lipid membranes, proteins, and DNA when present in high amounts. ROS release can induce two types of cell death; necrosis occurs at very high doses and when cells are exposed to ROS for long duration. On the other hand, ROS release causes apoptosis at comparably lower doses and therefore ROS release is being exploited as an anti-cancer mechanism [10].

Resveratrol is a stilbenoid that is found in the skin of red grapes and is known to suppress proliferation and induce apoptosis in a variety of cancer cells [11], [12]. Resveratrol has been shown to inhibit the activation of multiple dysregulated survival pathways including PI3-kinase/AKT pathway [13], [14] to induce apoptosis in various cancer cells. The exact mode of action of Resveratrol mediated anticancer effect is not fully understood. Several studies suggested that Resveratrol induces anti-neoplastic effect by reacting with cellular peroxidases and thiols thereby transforming into highly reactive phenoxyl radicals [15]. It has also been shown that Resveratrol at high concentrations leads to release of ROS in a variety of cells [16]. In addition, Resveratrol has been known to release ROS by several indirect pathways as well acting in association with NAD(P)H- dependent ROS production [17] or at the mitochondrial level [18]. Recently, it has also been shown that Resveratrol exerts its anti-proliferative action by arresting cells in the G1/G2 phase thereby inhibiting cell cycle progression [19].

In this study, we investigated whether Resveratrol inhibited cell viability and induced apoptosis via inactivation of AKT pathway in DLBCL cell lines. We have further extended our study to determine whether Resveratrol mediates its apoptotic effect via generation of ROS. We found that Resveratrol indeed induces apoptosis via inactivation of AKT through generation of ROS. In addition, Resveratrol treatment of DLBCL cells resulted in upregulation of DR5 through generation of ROS. This up-regulated DR5 secondary to Resveratrol treatment augmented DLBCL cells to low doses of TRAIL-induced apoptosis in these malignant cells. Based on these observations, Resveratrol–TRAIL combination may offer a new approach in effective treatment of DLBCL.

## Materials and Methods

### Cell lines

Sudhl4 and Sudhl10 cell lines were purchased from Deutsche Sammlung von Mikroorganismen und Zellkulturen (DSMZ), Braunschweig, Germany. HBL-1, OCI-LY3 and RIVA were a kind gift from Dr Laura Pasqualucci Institute for Cancer Genetics and the Herbert Irving Comprehensive Cancer Center, Columbia University, New York, USA. Sudhl4 and Sudhl10 cell lines were cultured in RPMI 1640 medium while HBL-1, OCI-LY3 and RIVA cell lines were grown in IMDM medium. All the experiments were performed in media containing 5% serum.

### Reagents and antibodies

Resveratrol was purchased from Calbiochem (Gibbstown, NJ). Bax (6A7) antibody, PEG-catalase and PEG-superoxide dismutase were purchased from Sigma (St. Louis MO, MA). Antibodies against p-AKT, caspase-9, p-FOXO-1, p-GSK3, p-Bad, cleaved caspase-3 and Bid were purchased from Cell Signaling Technologies (Beverly, MA, USA). Cytochrome c, beta-actin, caspase-3, SHP-TP1, p-Tyr and PARP antibodies were purchased from Santa Cruz Biotechnology, Inc. (Santa Cruz, CA, USA). XIAP, cIAP-1, cIAP-2, Survivin and caspase-8 antibodies were purchased from R&D (USA). Annexin V was purchased from Molecular Probes (Eugene OR, USA). Apoptotic DNA-ladder kit was obtained from Roche (Penzberg, Germany). H2DCFDA and JC1 dye were purchased from Alexis Corp (Farmingdale, NY, USA).

### 3-(4,5-Dimethylthiazol-2-yl)-2,5-Diphenyltetrazolium Bromide Assays

DLBCL cells were incubated at the concentration of 10^4^ cells in triplicates in a 96 well format. Cells were then treated with various doses of Resveratrol for 48 hours in a final volume of 0.2ml for 48 hours. Cell viability was measured by MTT cell viability assay, as previously described [5], [20]. 6 wells for each dosage including vehicle control were analyzed for each experiment. * denotes statistical significance.

### Live Dead Assay

To measure apoptosis, Live-Dead assay (Invitrogen, Eugene, OR) was used as described by the manufacturer. Briefly, 1×10^6^ DLBCL cells were treated with various doses of Resveratrol or TRAIL for 24 hours. Following incubation, cells were re-suspended in 1ml PBS containing 50 µM calcein AM and 8 µM ethidium homodimer and cells were incubated in the dark for 20 minutes. 50 µl of suspension was transferred on slides and visualized under an Olympus fluorescent microscope using a longpass filter.

### Cell cycle analysis, annexin V staining, and DNA laddering

DLBCL cell lines were treated with various concentrations of Resveratrol for 24 hours. For cell cycle analysis, cells were washed with PBS and re-suspended in 500μl hypotonic staining buffer and analyzed by flow cytometry as described previously [21]. For detection of apoptosis, cells were harvested and percentage apoptosis was measured by flow cytometry after staining with flourescein-conjugated annexin-V and propidium iodide (PI) (Molecular probes, Eugene, OR) and DNA laddering using a 1.5% agarose gel as described previously [22].

### Soft agar colony assays

Soft agar colony experiments were performed according to the manufacturer's protocol (Cheminon International, Temecula, CA). Briefly, following treatment with indicated doses of Resveratrol, 2500 cells were plated in 0.5ml culture medium containing 0.4% (v/v) top agar and layered over a basal layer of 0.8% (v/v) agar and 20% FBS with culture medium and allowed to grow for 4 weeks. Following 4 weeks incubation, cells were stained at a final concentration of 1mg/ml cell stain solution that was supplied with the kit.

### Measurement of ROS

We used H_2_DCFDA, a cell permeable fluorescent probe for detection for ROS release as described earlier [21]. Briefly, 1x10^6^ exponentially growing cells were loaded with 10 µM H2DCFDA for 45 minutes at 37°C and then treated with Resveratrol for various time periods in presence of 10mM NAC. Following incubation, the cells were washed with PBS and green fluorescence intensity in the cells was examined by FACS analysis.

### Cell lysis and Immunoblotting

For immunoblotting analysis, we extracted protein from Resveratrol treated cells as described previously [23]. Briefly, following treatment of DLBCL, cell were collected and were re-suspended in phosphorylation lysis buffer that contained 0.5–1.0% TritonX-100, 150mM NaCl, 1mM EDTA, 200μM sodium orthovanadate, 10mM sodium pyrophosphate, 100mM sodium fluoride, 1.5mM magnesium chloride, 1mmol/L phenylmethylsulfonyl-flouride, and 10μg/ml aprotonin. Bradford assay was used to assess protein concentrations and equal amount of proteins were separated by SDS-PAGE and transferred to polyvinylidene difluoride membrane (Immobilion, Millipore, etc). Immunoblotting was performed with different antibodies and visualized by an enhanced chemiluminescence (ECL, Amersham, Illinois, USA) method.

### Detection of Bax conformational changes

Detection of Bax conformation was performed as previously described [24]. In brief, cells were treated with Resveratrol for different time periods after which cells were harvested and washed with PBS and lysed with Chaps lysis buffer (10mM HEPES (ph 7.4), 150mM NaCl, 1% Chaps) containing protease inhibitors. Concentration of proteins was assessed by Bradford assay and 500 µg of total protein was incubated with 6 µg of anti-Bax 6A7 monoclonal antibody for 2 hours at 4°C. Following incubation, 25 µl of protein G-beads were added into the reaction and incubated at 4°C overnight on a shaker with gentle agitation. Following 4 washes in Chaps lysis buffer, samples were separated by SDS-PAGE, transferred and immunoblotted using N20 Bax polyclonal antibody.

### Assay for cytochrome c release

Release of cytochrome *c* from mitochondria was assayed as described earlier [24]. Briefly, After treatment of DLBCL cells with Resveratrol for 24 hours, cells were harvested, washed with PBS and cytosolic extracts were prepared and analyzed by immunoblotting using an anti-cytochrome c antibody.

### Measurement of mitochondrial potential using the JC-1 (5, 5′, 6, 6′-teterachloro-1, 1′, 3,3′- tetraethylbenzimidazolylcarbocyanine iodide) assay

1x106 cells were treated with Resveratrol for 24 hours after which the cells were washed with PBS and re-suspended in mitochondrial incubation buffer (Alexis Corporation, Farmingdale, NY, USA). JC1 (Alexis corp) was then added to the cells at a final concentration of 10μM and incubated at 37°C in dark for 30 minutes and mitochondrial membrane potential (% of green and red aggregates) was determined by flow cytometry as described previously [25].

### In vitro kinase assay

AKT kinase assay (Abcam, ab65786, Cambridge, MA, USA) was performed according to the manufacturer's protocol. Briefly, DLBCL cells were treated with various doses of Resveratrol for 24 hours. Following treatment, cells were pelleted (10–15×10^6^) and washed once with ice cold PBS and lysed in ice cold kinase extraction buffer. For each assay, 2 µg of AKT specific antibody and protein A beads were added to 200 µg proteins and incubated overnight at 4°C with gentle agitation. Next day, cells were pelleted and washed with kinase extraction buffer and once with kinase assay buffer. 50 µl Kinase Assay Buffer and 2 µl GSK-3 alpha Protein/ATP Mixture was added to the protein A beads and incubated at 30°C for 4 hours. After incubation, protein A beads were spun, boiled and separated by SDS-PAGE, transferred to polyvinylidene difluoride membrane and probed with p-GSK3 antibody.

### RT-PCR assays

Total RNA was extracted following treatment with 25 and 50 µM apigenin for 24 hours using TRIZOL and reverse-transcribed with random hexamers. RT-PCR amplifications were performed using the following primers:

**AKT1-For:** GCTGGACGATAGCTTGGA

**AKT1-Rev:** GATGACAGATAGCTGGTG,

**AKT2-For:** GGCCCCTGATGAGACTCTA,

**AKT2-Rev:** TCCTCAGTCGTGGAGGAGT, and

**AKT3-For:** GCAAGTGGACAGGAATAAGTCTC,

**AKT3-Rev:** ACAATGGTGGGCTCATGACTTCC

for 35 cycles (55°C annealing temperature) to yield 382bp, 275bp and 328bp products respectively. Amplification of GAPDH was used as an internal control.

### Gene Silencing using SiRNA

DR5 siRNA (cat no. S100056707 and cat no. S100056700 pooled) and Scrambled control siRNA (cat no. 102781) were purchased from Qiagen. The method used for transfection was the same as previously described [5]. In brief, cells were washed with serum free media and re-suspended in a complex containing LipofectAMINE 2000 reagent (Invitrogen, Carlsbad, CA) and the desired siRNA for 6hours. After incubation, the lipid and siRNA complex was removed and fresh growth medium was added. Cells were treated 48 hours after transfection for 24 hours and specific protein levels were determined by Western Blot analysis with specific antibodies against the targeted proteins and actin as a loading control.

### Statistical Analysis

Data are presented, as mean ± SD. Comparisons between groups were made with the paired Student's t-test. Values of p<0.05 were considered statistically significant.

## Results

### Resveratrol treatment causes inhibition of viability and induces apoptosis in DLBCL cell lines

**Figure 1:**
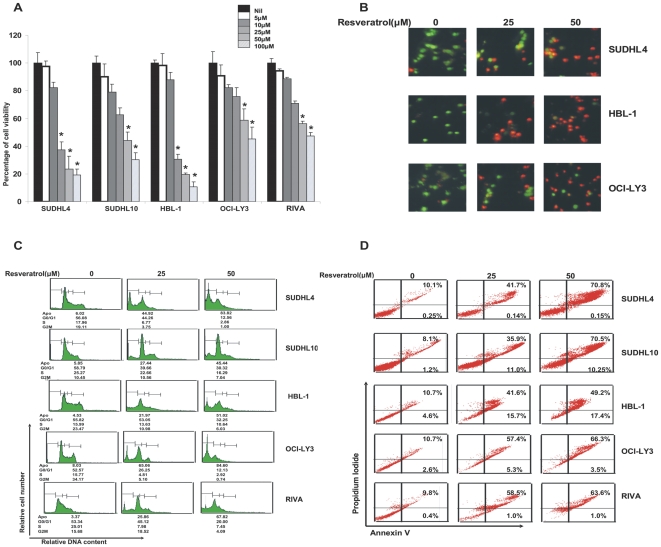
Resveratrol suppresses growth and induces apoptosis in DLBCL cells. (**A**) DLBCL cell lines were incubated with 0–100 µM Resveratrol for 24 hours. Cell viability was measured by MTT assays as described in Materials and Methods. The graph displays the mean +/− SD (standard deviation) of three independent experiments, * p<0.05, statistically significant (Students *t*-test). (**B**) SUDHL4, HBL-1 and OCILY3 cells were treated with 25 and 50 µM Resveratrol for 24 hours and apoptosis was measured by Live/Dead Assay. (**C**) DLBCL cells were treated with 25 and 50 µM Resveratrol for 24 hours. Thereafter, the cells were washed, fixed and stained with propidium iodide, and analyzed for DNA content by flow cytometry as described in “Materials and Methods”. (**D**) DLBCL cells were treated with 25 and 50 µM Resveratrol (as indicated) for 24 hours and cells were subsequently stained with flourescein-conjugated annexin-V and propidium iodide (PI) and analyzed by flow cytometry.

Resveratrol has been previously shown to inhibit cell viability and induce apoptosis in various cancers [26], [27]; therefore, we initially sought to determine whether Resveratrol induced inhibition of cell viability and induced apoptosis in DLBCL cells. DLBCL cells were treated with 5, 10, 25, 50 and 100 µM Resveratrol for 48 hours and analyzed for cell viability by MTT assays. As shown in Figure 1A, there was dose dependent inhibition in cell viability in all cell lines tested and this inhibition reached statistical significance for most of the doses. We then examined cells under a microscope after staining them with calcein and ethidium homodimer to check for plasma membrane integrity following treatment with 25 and 50 µM Resveratrol for 24 hours. As shown in Figure 1B, untreated cells were stained green depicting alive cells with plasma membrane integrity intact while cells treated with Resveratrol showed an increase of red cells suggesting disruption of plasma membrane integrity, i.e., dead cells. Next we treated DLBCL cells with 25 and 50 µM Resveratrol for 24 hours and determined the cell cycle fractions by flow cytometry. Following treatment with Resveratrol, there was an increase in the subG1/Apo fraction of cells from 6.02% to 44.92% and 83.82% after treatment with 25 µM and 50 µM Resveratrol in SUDHL4 cell line. Similar results were obtained in other DLBCL cell lines (Figure 1C). We analyzed Resveratrol treated DLBCL cell lines for apoptosis after FITC-conjugated annexin V and propidium iodide (PI) staining by flow cytometry. As shown in Figure 1D, we found that SUDH4 cell line had 42% and 71% apoptosis, SUDHL10 (47% and 80%), HBL-1 (57% and 66%), OCI-LY3 (62% and 69%) and RIVA (59% and 64%) after 25 and 50 µM Resveratrol treatment respectively. These data were finally confirmed by DNA laddering test that showed fragmentation of chromosomal DNA into 180bp bands in SUDHL4 and HBL-1 following treatment with 25 and 50 µM Resveratrol (Figure S1A). We also performed soft agar colony assays following treatment with 25 and 50 µM Resveratrol for 4 weeks. As shown in Figure S1B and C, Resveratrol treatment inhibited colony formation in HBL-1 cell line. These set of data clearly demonstrated that Resveratrol treatment inhibited cell viability and induced apoptosis in DLBCL cell lines.

### Resveratrol treatment causes in-activation of AKT and its down-stream targets in DLBCL cells

**Figure 2:**
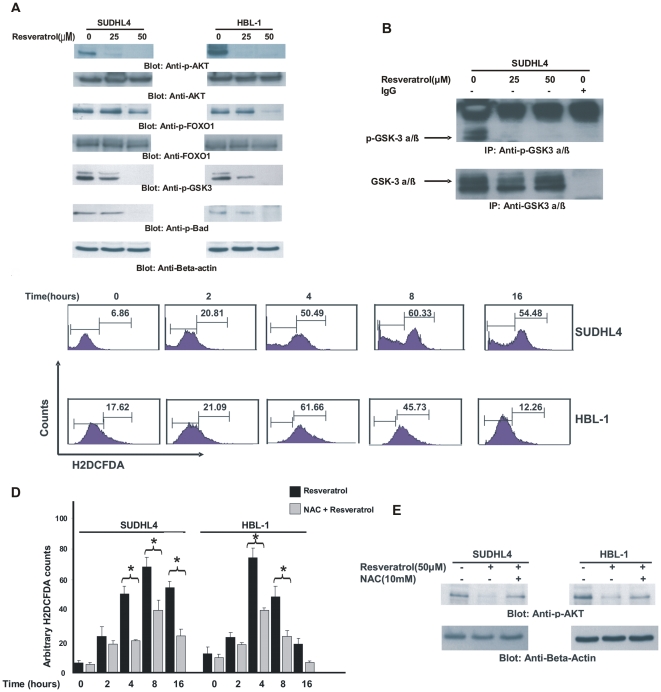
Resveratrol inhibits constitutive active AKT and its downstream effectors in DLBCL cells. (**A**) SUDHL4 and HBL-1 cells were treated with various doses of Resveratrol. Equal amount of protein from each sample was immunoblotted with Phospho-Akt-Ser473, total AKT Phospho-FOXO1, total FOXO1, p-GSK3, Phospho-Bad and Beta-actin. (**B**) SUDHL4 cells were treated with 25 and 50 µM Resveratrol for 24 hours. Cells were lysed and immunoprecipitated with either AKT antibody or IgG along with protein A beads overnight. After incubation, beads were washed and incubated with kinase buffer and 2 µl GSK-3 Protein/ATP mixture at 30°C for 4 hours. Beads were boiled and equal amount of protein was immunoblotted with antibody against p-GSK3. (**C**) SUDHL4 and HBL-1 cells were loaded with 10 µM H2DCFDA for 45 minutes and then were incubated with 25 µM Resveratrol for indicated time periods. Cells were re-suspended in PBS and analyzed for intracellular accumulation of H2DCFDA by flow cytometry (**D**) SUDHL4 and HBL-1 cell lines were loaded with 10 µM H2DCFDA for 45 minutes and then were pre-treated with 10mM NAC for 2 hours followed by treatment with 50 µM Resveratrol for various time periods. Cells were re-suspended in PBS and analyzed using flow cytometry. Bar graph displays the mean +/− SD (standard deviation) of three independent experiments, * p<0.05, statistically significant (Students *t*-test). (**E**) SUDHL4 and HBL-1 cells were pretreated with 10mM NAC for 2 hours followed by treatment with 50 µM Resveratrol for 24 hours. After cell lysis, equal amounts of proteins were immunoblotted with antibodies against p-AKT and beta actin as indicated.

Resveratrol has been shown to disrupt various survival pathways including PI3-kinase/AKT pathway in various cancers [28], [29]. For this reason, we sought to determine whether Resveratrol was inducing pro-apoptotic action via in-activation of PI3-kinase/AKT activity in DLBCL cells. SUDHL4 and HBL-1 cell lines were treated with 25 and 50 µM Resveratrol for 24 hours and proteins were extracted and immuno-blotted with antibodies against activated-AKT, total AKT and its downstream targets. As shown in Figure 2A, constitutive activation of AKT was in-activated following treatment with Resveratrol in both the cell lines; however, there was no effect on expression of total AKT. In addition, Resveratrol treatment of PEL cells did not affect the transcript levels of AKT1, AKT2 and AKT3 as well when detected by RT-PCR (Figure S2A). Forkhead Box-O (FOXO-1), a downstream target of AKT is emerging as a key regulator of cell survival in various cancers [30]. Resveratrol treatment of DLBCL cells also in-activated FOXO-1 without affect the expression of total Foxo-1 protein in a dose dependent manner in both the cell lines. GSK-3 is another down-stream target of AKT and plays an important role in cell survival [31]. Resveratrol treatment also caused in-activation of GSK-3 in DLBCL cell lines. In addition, we also determined whether Resveratrol in-activated phosphorylation of Bad, another target of AKT that plays an important role in activating the mitochondrial apoptotic pathway. As shown in Figure 2A, Resveratrol treatment also caused in-activation of Bad in both the cell lines. Finally, we assessed whether Resveratrol inhibited AKT kinase activity in DLBCL cells. SUDHL4 cells were treated with 25 and 50 µM Resveratrol for 24 hours and cell lysates were immunoprecipitated with an anti-AKT antibody or rabbit IgG, and *in vitro* kinase assays were performed as described in material and method section. Resveratrol treatment resulted in inactivation of AKT activity and dephosphorylation of GSK3 in SUDHL4 cells (Figure 2B). This data suggests that Resveratrol treatment causes inhibition of kinase activity of AKT in DLBCL cells.

### Resveratrol causes release of ROS in DLBCL cell lines

The exact mode of action of Resveratrol is not actually known, however, it has been suggested that Resveratrol acts via release of ROS [13], [14]. Therefore, it became pertinent to us to detect whether Resveratrol treatment of DLBCL cells was causing ROS release. SUDHL4 and HBL-1 cells were therefore loaded with H2DCFDA and then treated with 50 µM Resveratrol for various time periods and cells were analyzed by flow cytometry. As shown in Figure 2C, Resveratrol treatment caused release of ROS, as early as 2 hours that continued up to 8 hours of treatment in SUDHL4 and HBL-1 cell lines. This data was confirmed by pre-treating the cells with NAC, a scavenger of ROS for 2 hours followed by treatment with Resveratrol. As shown in Figure 2D, NAC pre-treatment significantly abrogated ROS release in both cell lines. To further confirm whether ROS release plays a major role in inducing apoptosis in DLBCL cells, we also pre-treated DLBCL cells with either PEG-catalase or PEG-superoxide dismutase (SOD) for 2 hours followed by treatment with 50 µM Resveratrol for 24 hours. As shown in Figure S1D, PEG-catalase and PEG-SOD pre-treatment significantly inhibited Resveratrol induced apoptosis in DLBCL cells.

### Resveratrol in-activates AKT via release of ROS

To better understand the role of ROS release in in-activation of AKT, we pre–treated DLBCL cells with 10mM NAC for 2 hours followed by treatment with Resveratrol for 24 hours. Proteins were prepared and immuno-blotted with antibodies against p-AKT and Beta-actin. As shown in Figure 2E, Resveratrol treatment in-activated AKT but pre-treatment with NAC prevented Resveratrol-induced in-activation of AKT. This data clearly demonstrates that Resveratrol treatment causes release of ROS that, in turn, causes in-activation of AKT. The exact mechanism by which ROS release leads to in-activation of AKT is not known. However, there are reports that suggest that AKT can be de-phosphorylated by various phosphatases such as PTEN and SHPTP1 [32], [33], [34], [35]. We found that PTEN expression was not altered by Resveratrol treatment (Data not shown) of DLBCL cells suggesting that PTEN is not involved in Resveratrol-mediated in-activation of AKT. SHP-TP1, a tyrosine phosphatase has been shown to regulate the activity of AKT [35]. Therefore, we next determined whether Resveratrol treatment could modulate the activity of SHP-TP1 in DLBCL cells. SUDHL4 cells were treated with either 25 or 50 µM Resveratrol alone or in the presence of NAC. Following treatment, cells were lysed and immunoprecipitated with either SHP-TP1 antibody or rabbit IgG as indicated in Figure S2B. Proteins were separated on SDS-Page and immunoblotted with antibody against p-Tyrosine. Our data indicated that Resveratrol treatment led to activation (phosphorylation) of SHP-TP1 in a dose dependent manner and this activation was blocked by pre-treatment L with NAC. These data suggests that Resveratrol-mediates the induction of SHP-TP1 activity in a ROS dependent manner in DLBCL cells.

### Resveratrol treatment causes activation of mitochondrial apoptotic pathway and release of cytochrome c into cytosole in DLBCL cells

**Figure 3:**
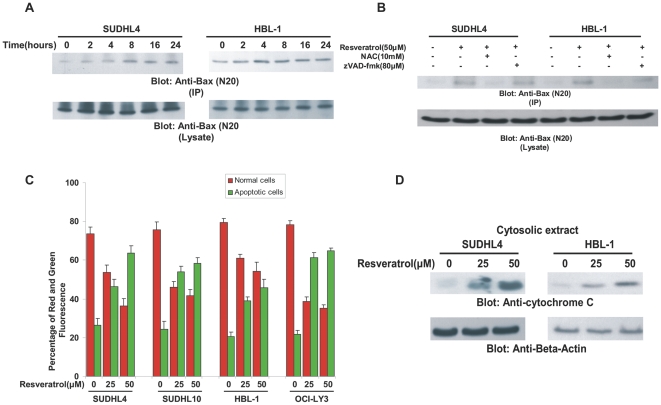
Resveratrol-induced mitochondrial signaling pathway in DLBCL cells. (**A**) After treating with 50 µM Resveratrol for indicated time periods, HBL-1 and SUDHL4 cells were lysed and immuno-precipitated with anti-Bax 6A7 antibody for detection of conformationally changed Bax protein. In addition, the total cell lysates were immuno-blotted with specific anti-Bax polyclonal antibody. (**B**) HBL-1 and SUDHL4 cells were pre-treated with either, 10mM NAC and 80 µM z-VAD/fmk for 2 hours and subsequently treated with 50 µM Resveratrol for 8 hours. Cells were lysed and immunoprecipitated with anti-Bax 6A7 antibody and proteins were immunoblotted with Bax rabbit polyclonal antibody. (**C**) DLBCL cells were treated with and without 25 and 50 µM Resveratrol for 24 hours. Live cells with intact mitochondrial membrane potential and dead cells with lost mitochondrial membrane potential was measured by JC-1 staining and analyzed by flow cytometry as described in Materials and Methods. (**D**) SUDHL4 and HBL-1 cells were treated with 25 and 50 µM Resveratrol for 24 hours. Mitochondrial free cytosolic fractions were isolated and immunoblotted with antibody against cytochrome c and Beta-actin.

In-activation of Bad has been shown to activate the mitochondrial apoptotic pathway by allowing Bad protein to translocate to mitochondria leading to up-regulation of pro-apoptotic Bax [36]. To examine whether this theory hold true for Resveratrol-induced activation of mitochondrial apoptotic pathway, we treated SUDHL4 and HBL-1 cell lines with 50 µM Resveratrol for various time periods and then examined the conformational changes in Bax protein by immuno-precipitation. We found evidence that Bax protein underwent conformational changes, as early as 2 hours in HBL-1 cell line and 4 hours in SUDHL4 cell line (Figure 3A). To confirm whether Bax conformational changes was due to ROS release and not due to activation of caspases, we pre-treated HBL-1 cells with either 10mM NAC or 80 µM zVAD-fmk for 2 hours followed by treatment with 50 µM Resveratrol for 8 hours and Bax conformation changes were detected by immuno-blotting. As shown in Figure 3B, Bax conformational changes were blocked only in NAC pre-treated cells following treatment with Resveratrol. This data suggested that ROS release is required for activation of Bax protein in DLBCL cell lines treated with resveratrol. We next treated all the DLBCL cell lines with 25 and 50 µM Resveratrol for 24 hours and then stained the cells with JC1 and analyzed the cells by flow cytometry and found that DLBCL cells treated with Resveratrol had an increase in green stained apoptotic cells as compared to normal cells that stained red (Figure 3C) confirming change in mitochondrial membrane potential. Once there are changes in the mitochondrial membrane potential, it leads to release of cytochrome c into cytosole. To assess this, we prepared mitochondrial free cytosolic extracts of SUDHL4 and HBL-1 cell lines treated with Resveratrol and immuno-blotted them with antibody against cytochrome c. As shown in Figure 3D, the cytosolic fraction showed an increase in cytochrome c expression following treatment with Resveratrol in both, SUDHL4 and HBL-1 cell lines. This data clearly suggests that Resveratrol treatment caused release of cytochrome c in DLBCL cells. Inhibitor of apoptosis proteins (IAPs) plays a very important anti-apoptotic role by inhibiting the activation of caspase-9 and/or caspase-3 thereby preventing the apoptotic signal to proceed. Therefore, we wanted to examine whether Resveratrol treatment of DLBCL cells down-regulated the expression of these proteins as well. Resveratrol treatment of DLBCL caused down-regulation of IAP family members, XIAP, cIAP1 and Survivin in a dose dependent manner (Figure S3).

### Caspases activation is essential for Resveratrol-induced apoptosis in DLBCL

**Figure 4:**
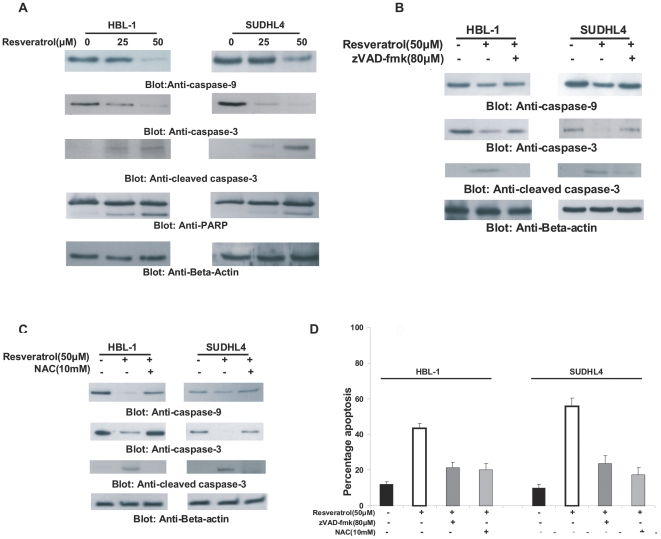
Activation of caspases -9, -3 and cleavage of PARP induced by Resveratrol treatment in DLBCL cells. (**A**) SUDHL4 and HBL-1 cells were treated with and without 25 and 50 µM Resveratrol for 24 hours. Cells were lysed and equal amounts of proteins were immunoblotted with antibodies against caspase-9, caspase-3, cleaved caspase-3, PARP and Beta-actin. SUDHL4 and HBL-1 cells were pretreated with either 80 µM z-VAD (**B**) or 10mM NAC (**C**) for 2 hours and subsequently treated with 50 µM Resveratrol for 24 hours. Cells were lysed and equal amounts of proteins were immunoblotted with antibodies against caspase-9, caspase-3 cleaved caspase-3 and beta-actin. (**D**) SUDHL4 and HBL-1 cells were pretreated with either 80 µM of z-VAD-fmk or 10mM NAC for 2 hours and subsequently treated with 50 µM Resveratrol for 24 hours. Following treatment, cells were stained with fluorescein conjugated annexinV/PI and apoptosis was measured by flow cytometry.

For efficient apoptosis to occur, the caspases family of proteins needs to be activated and cleaved, whether the apoptotic signal is initiated by the extrinsic or intrinsic apoptotic pathway. To analyze the caspases, we treated SUDHL4 and HBL-1 cells with 25 and 50 µM Resveratrol for 24 hours and assessed for caspase activation and cleavage by immuno-blotting. As shown in Figure 4A, caspases-9 and -3 were activated and cleaved followed by cleavage of PARP in both the cell lines. To confirm whether caspase activation is necessary for induction of apoptosis, we pre-treated DLBCL cells with a universal inhibitor of caspases, zVAD-fmk for 2 hours followed by 24 hour treatment of Resveratrol. zVAD-fmk pre-treatment inhibited Resveratrol-induced activation of caspases-9, -3 and cleavage of PARP (Figure 4B). In addition, we also wanted to know whether ROS release played any role in activation and cleavage of caspases. Therefore, we pre-treated DLBCL cells with 10mM NAC for 2 hours followed by treatment with Resveratrol for 24 hours. As seen in Figure 4C, NAC pre-treatment prevented Resveratrol-induced activation of caspases-9 and -3 and cleavage of PARP. In addition, DLBCL cells pre-treated with either zVAD-fmk or NAC also prevented Resveratrol-induced apoptosis (Figure 4D). These set of data suggest that caspases activation is essential for Resveratrol-induced apoptosis however Resveratrol induces its apoptotic action via release of ROS.

### Resveratrol-induced release of ROS causes up-regulation of DR5 in DLBCL cells

**Figure 5:**
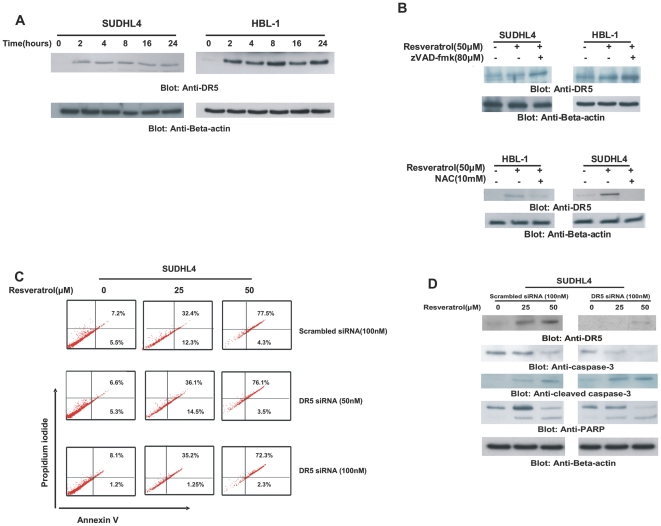
Reservatrol-mediated upregulation of DR5. (**A**) SUDHL4 cells were treated with 50 µM Resveratrol for indicated time periods. After cell lysis, equal amounts of proteins were immuno-blotted with antibodies against DR5 and beta actin. (**B**) SUDHL4 and HBL-1 cells were pre-treated with either 80 µM of z-VAD (upper panel) or 10mM NAC (lower panel) for 2 hours and subsequently treated with 50 µM Resveratrol for 24 hours. Cells were lysed and equal amounts of proteins were immunoblotted with antibodies against DR5 and beta-actin. (**C**) SUDHL4 cells were either transfected with 50 and 100nM siRNA, specific against DR5 or scrambled siRNA for 48 hours. Cells were then treated with 25 and 50 µM Resveratrol for 24 hours, following which cells were stained with fluorescent-conjugated Annexin V/PI and analyzed by flow cytometry. (**D**) SUDHL4 cells were either transfected with 100nM siRNA, specific against DR5 or scrambled siRNA for 48 hours and treated with 25 and 50 µM Resveratrol for 24 hours. Cells were lysed and equal amounts of proteins were immuno-blotted with antibodies against DR5, caspase-3, cleaved caspase-3, PARP and beta-actin.

Tumor necrosis factor related apoptosis inducing ligand (TRAIL) has the ability to selectively kill cancer cell, however, resistance to TRAIL quickly develops in cancer cells [37]. Death Receptor 5 (DR5) has been shown to be up-regulated by ROS release [21], therefore, we sought to determine whether Resveratrol-induced release of ROS caused up-regulation of DR5 in DLBCL cells. SUDHL4 and HBL-1 cells were treated with 50 µM Resveratrol for various time periods and proteins were separated on SDS-Page and immuno-blotted with antibody against DR5. Resveratrol treatment caused up-regulation of DR5 within 4hours and continued to be up-regulated up to 24 hours (Figure 5A). We next pre-treated DLBCL cells with either 80 µM zVAD-fmk or 10mM NAC for two hours followed by treatment with 50 µM Resveratrol for 24 hours. As shown in Figure 5B (upper panel), zVAD-fmk pre-treatment did not inhibit Resveratrol-induced up-regulation of DR5 while NAC pre-treatment blocked DR5 up-regulation Figure 5B (lower panel) clearly suggesting that DR5 up-regulation does not depend upon caspase-activation but on ROS release.

### DR5 does not play a role in Resveratrol-induced apoptosis in DLBCL cells

TRAIL has the ability to induce apoptosis through interacting with its death receptors [38]. It has also been shown that up-regulation of DR5 alone is not sufficient to induce apoptosis and requires its ligand, TRAIL for efficient apoptosis to occur [39]. We therefore sought to determine whether DR5 up-regulation alone plays an active role in Resveratrol-induced apoptosis in DLBCL cells. Therefore, we transfected SUDHL4 and HBL-1 cells with siRNA against DR5 for 48 hours and then treated the cells with 25 and 50 µM Resveratrol for 24 hours. As shown in Figure 5C, DR5 knockdown of DLBCL cells did not prevent Resveratrol-induced apoptosis as measured by annexin V/PI dual staining analyzed by flow cytometry. In addition, caspase -3 was also activated and PARP was cleaved in DR5 knockdown cells treated with Resveratrol (Figure 5D). This data suggests that even though, Resveratrol has the ability to up-regulate DR5, this up-regulation does not play a role in Resveratrol-induced apoptosis in DLBCL cells.

### Resveratrol and TRAIL induces synergistically potent apoptosis in DLBCL cells

**Figure 6:**
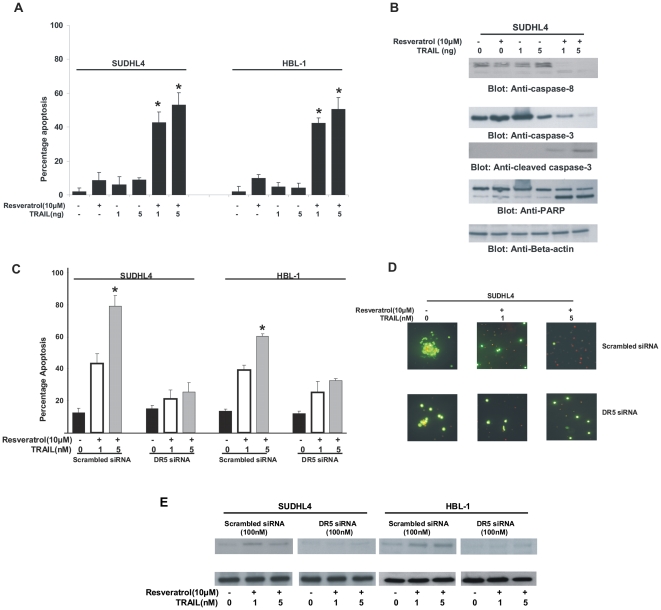
Resveratrol treatment potentiates TRAIL mediated apoptosis in DLBCL cells. (**A**) SUDHL4 and HBL-1 cells were treated with either 10 µM Resveratrol in the presence and absence of 1 and 5ng TRAIL for 24 hours. Following treatment, cells were stained with fluorescent-conjugated Annexin V/PI and analyzed by flow cytometry. (**B**) SUDHL4 cells were treated with either 10 µM Resveratrol in the presence and absence of 1 and 5ng TRAIL for 24 hours. Following treatment, cells were lysed and equal amounts of proteins were immuno-blotted with antibodies against caspase-8, caspase-3, PARP, and beta- actin. (**C**) SUDHL4 and HBL-1 cells were transfected with either scrambled siRNA (100nM) or DR5 specific siRNA (100nM) for 48 hours and then cells were treated with 10 µM Resveratrol in the presence of either 1 or 5ng TRAIL for 24 hours following which cells were stained with fluorescent-conjugated Annexin V/PI and analyzed by flow cytometry, * denotes statistical significance (p<0.05) or (**D**) cells were stained with 50 µM calcein AM and 8 µM ethidium homodimer and visualized under an Olympus fluorescent microscope using a long-pass filter. (E) SUDHL4 and HBL-1 cells were transfected with either scrambled siRNA (100nM) or DR5 specific siRNA (100nM) for 48 hours and then cells were treated with 0 µM Resveratrol in the presence of either 1 or 5ng TRAIL for 24 hours following which cells were lysed and immunoblotted with antibodies against DR5 and Betaactin.

In order to utilize up-regulation of DR5 effectively to induce a more potent apoptosis, we treated DLBCL cell lines with a combination of sub-toxic doses of Resveratrol (10 µM) and TRAIL (1 and 5ng) for 24 hours and assessed the apoptotic response. As shown in Figure 6A, neither Resveratrol nor TRAIL at sub-toxic doses could induce apoptosis alone, however, when both the drugs were given in combination, there was efficient apoptosis in SUDHL4 and HBL-1 cell lines. In addition, combination of Resveratrol and TRAIL was also able to activate caspase-8, caspase-3 and cleave PARP (Figure 6B). To confirm these findings, we knocked down the expression of DR5 using specific siRNA against DR5 followed by combination treatment of sub-toxic doses of Resveratrol and TRAIL for 24 hours in DLBCL cells. As expected, we found that DLBCL cells that were transfected with scrambled non-specific siRNA showed a synergistic apoptotic response to the combination treatment; however, in those cells that were transfected with siRNA against DR5, there was a diminished apoptotic response following combination treatment of Resveratrol and TRAIL at sub-toxic doses (Figure 6C and D). In addition, immuno-blot analysis also showed that there was up-regulation of DR5 in SUDHL4 and HBL-1 cells transfected with scrambled siRNA following Resveratrol treatment, however, the expression of DR5 in DLBCL cells transfected with siRNA against DR5 was diminished or absent even after treatment with Resveratrol (Figure 6E). These data suggest that Resveratrol augmentation of TRAIL induced apoptosis occurs via up-regulation of DR5.

## Discussion

Induction of apoptosis in malignant cells is a very important mechanism of action of anticancer drugs [40]. Resveratrol (3,5,4′-trihydroxy-trans-stilbene) is a stilbenoid, produced naturally by several plants has been shown to have anticancer activity in vitro and animal studies [11], [26], [27]. We now provide evidence that Resveratrol induces cell death and apoptosis in a panel of diffuse large B cell lymphoma (DLBCL) cell lines. We have recently shown that activated AKT was present in 52% of DLBCL tumor cells [6]. Furthermore, high p-AKT expression was associated with short survival, thereby suggesting that the PI3K/AKT pathway may be a potential target for therapeutic intervention in DLBCL**.** In this study, we found that Resveratrol induces its pro-apoptotic effects via inactivation of AKT and its downstream targets; FOXO1, GSK3 and Bad.

The exact mechanism of action of Resveratrol is not fully understood, however, it has been proposed that Resveratrol causes its pro-apoptotic effects via generation of ROS in various cancers [13], [14]. We confirm these findings by clearly demonstrating by H2DCFDA based experiments that Resveratrol treatment causes release of ROS in DLBCL cells. Furthermore, we also show that Resveratrol-induced inactivation of AKT is also ROS release dependent as pre-treatment of DLBCL cells with N-acetyl cysteine (NAC), a scavenger of ROS inhibited Resveratrol-induced inactivation of AKT. Two additional ROS scavengers, PEG-catalase and PEG-SOD were also used to confirm the role of ROS release in inducing apoptosis in DLBCL. The exact mechanism by which Resveratrol-induced ROS release leading to in-activation of p-AKT is not known. Our data suggests that Resveratrol-induced ROS release activate the tyrosine phosphatase, SHP-TP1 resulting in de-phosphorylation of AKT. Therefore, these finding implicate that Resveratrol inhibits AKT activity via modulation of SHP-TP1 through the release of ROS in DLBCL cells.

The mitochondrial apoptotic pathway plays an important role on inducing apoptosis via activation of pro-apoptotic molecules such as Bax leading to release of cytochrome c into cytosole and activation of caspases [41]. Resveratrol mediated its apoptotic effects in DLBCL cells via in-activation of Bad leading to conformational changes in Bax protein and its translocation into the mitochondrial membrane. Once Bax is translocated to the mitochondrial membrane, it renders the membrane leaky thereby causing changes in the mitochondrial membrane potential. Loss of mitochondrial membrane potential is one of the main mechanisms responsible for cytochrome c release in response to different cytotoxic stimuli. In cytosol, cytochrome c plays a key role by activating pro-caspase 9 in the presence of ATP. This in turn causes cleavage of caspase-9 that propagates the death signal by activating caspase-3 and causing cleavage of PARP. Activation and cleavage of PARP is the hallmark of apoptosis that in turn causes DNA fragmentation and cell death.

There are studies that have shown that ROS release sensitizes cancer cells to TRAIL induced apoptosis via up-regulation of DR5 in various cancers [42], [43], [44], [45], [46]. In concordance with these studies, our data also showed that Resveratrol treatment of DLBCL cells caused up-regulation of DR5 via generation of ROS. However, up-regulation of DR5 did not play a role in inducing apoptosis in these cell lines. We confirmed these finding by knocking down expression of DR5 in DLBCL cell lines, Resveratrol was still able to induce efficient apoptosis as was evident by flow cytometry results and immuno-blotting that showed activation of caspases and cleavage of PARP in Resveratrol-treated DLBCL cells. Horndasch et al have recently shown that Resveratrol sensitized prostate cancer cells to TRAIL-induced apoptosis [47]. In concordance to this study, we were also able to synergize DLBCL cells with Resveratrol to TRAIL-induced apoptosis. Indeed, up-regulation of DR5 by Resveratrol treatment does give an added attractive target to induce more potent apoptosis without causing toxicity by using combination treatment with sub-toxic doses of Resveratrol and TRAIL. These experiments allow us to utilize the intrinsic as well as the extrinsic apoptotic pathway to induce efficient apoptosis. The upregulation of DR5 by Resveratrol hence sensitizes DLBCL cells to TRAIL-induced apoptosis and therefore has potential clinical application in the management of B-cell malignancies. To our knowledge, this is the first report on the ability Resveratrol to augment TRAIL's apoptotic effects via upregulation of DR5 in DLBCL.

**Figure 7:**
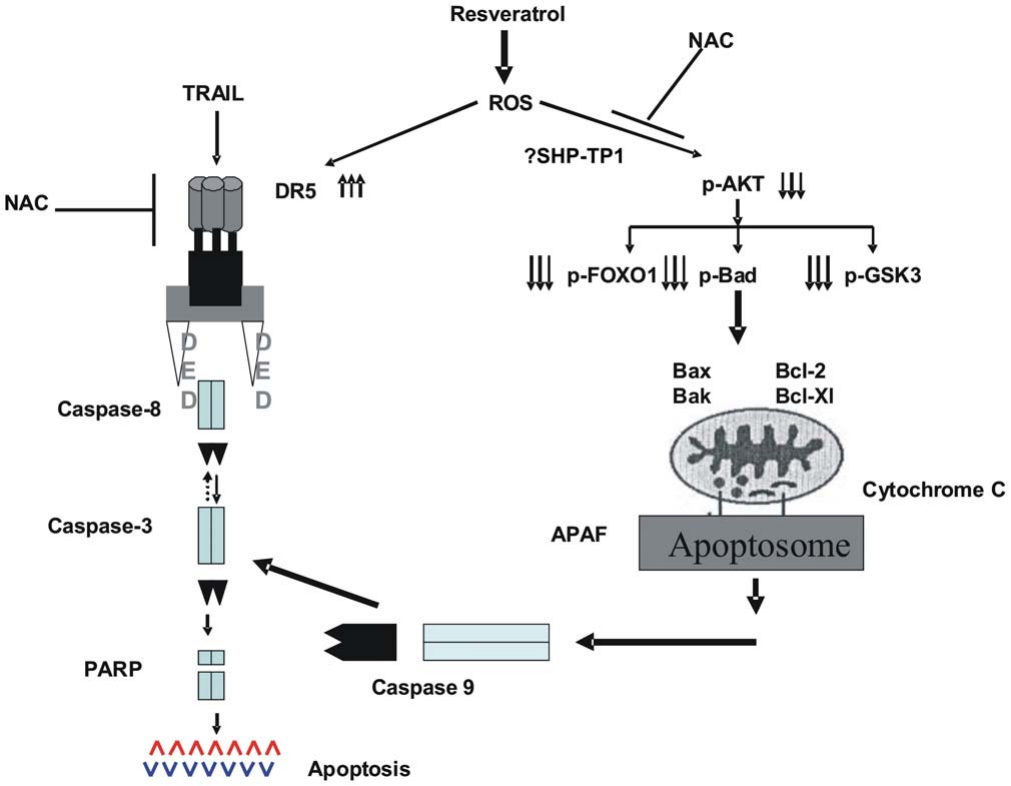
Schematic representation of Resveratrol-induced apoptosis in DLBCL.

In summary our findings show (Schematic proposed in Figure 7) that Resveratrol-induced apoptosis occurs via release of ROS. ROS release led to in-activation of AKT and its down-stream targets; FOXO1, GSK3 and Bad. Once Bad is in-activated, it allowed conformational changes in Bax protein leading to change in mitochondrial membrane potential, release of cytochrome c into cytosole and apoptosis via the intrinsic apoptotic pathway. In addition, ROS release also caused up-regulation of DR5 and co-treatment of DLBCL cells with Resveratrol and TRAIL significantly enhanced apoptosis in DLBCL. These data demonstrates a viable strategy of therapeutic intervention for the management of DLBCL.

## Supporting Information

Figure S1 **Anticancer effects of Resveratrol on DLBCL cells.** (**A**) HBL-1 and SUDHL4 cells were treated with 25 and 50 µM Resveratrol for 24 hours and DNA was extracted and separated by electrophoresis on 1.5% agarose gel. (**B**) Clonogenic assays were performed as described in Materials and Methods. BC1 cells were treated with 25 and 50 µM Resveratrol for 24 hours. Subsequently, cells were plated in Soft agar plates for 4 weeks. Cells were stained and manually counted. (**C**) The bar graph displays the mean ± standard deviation of three independent experiments. * denotes statistically significant students ttest (p<0.05). **(D)** HBL-1 and SUDHL4 cells were treated with 50 µM Resveratrol for 24 hours in the presence or absence of pretreatment with PEG-catalase and PEG-superoxide dismutase for 2 hours. Following treatment, cells were stained with fluorescen-conjugated annexin V/PI and cells were analyzed by flow cytometry. Bar graph denotes a mean of three independent experiments.
(TIF)

Figure S2 **Effect of Resveratrol on AKT expression and SHP-TP1 activivity. **(**A**) 5x106 cells were treated with and without indicated doses of Resveratrol for 24 hours. RNA was isolated, and reverse transcribed as described in material and methods. Block RT-PCR for AKT1, AKT2 and AKT3 were performed for 35 cycles at 55°C. GAPDH was used as an internal control. (**B**) SUDHL4 cells were treated with either 25 and 50 µM Resveratrol or NAC and Resveratrol for 24 hours and cells were immuno-precipitated with SHP-TP1 antibody. Proteins were separated on SDS-Page and immuno-blotted with p-Tyrosine antibody and SHP-TP1 antibody.
(TIF)

Figure S3 **Resveratrol down-regulates expression of IAPs in DLBCL.** HBL-1 and SUDHL4 cells were treated with 25 and 50 µM Resveratrol for 24 hours. Following incubation, cells were harvested and proteins were isolated that were separated on SDS-Page and immunoblotted with antibodies against XIAP, cIAP1, Survivin and beta-actin as indicated.
(TIF)
