# Supplementary figures and images for: Resveratrol Suppresses Constitutive Activation of AKT via Generation of ROS and Induces Apoptosis in Diffuse Large B Cell Lymphoma Cell Lines
Source: PLoS One. 2011 Sep 12;6(9):e24703. doi: 10.1371/journal.pone.0024703 (PMC3171480; doi:10.1371/journal.pone.0024703)

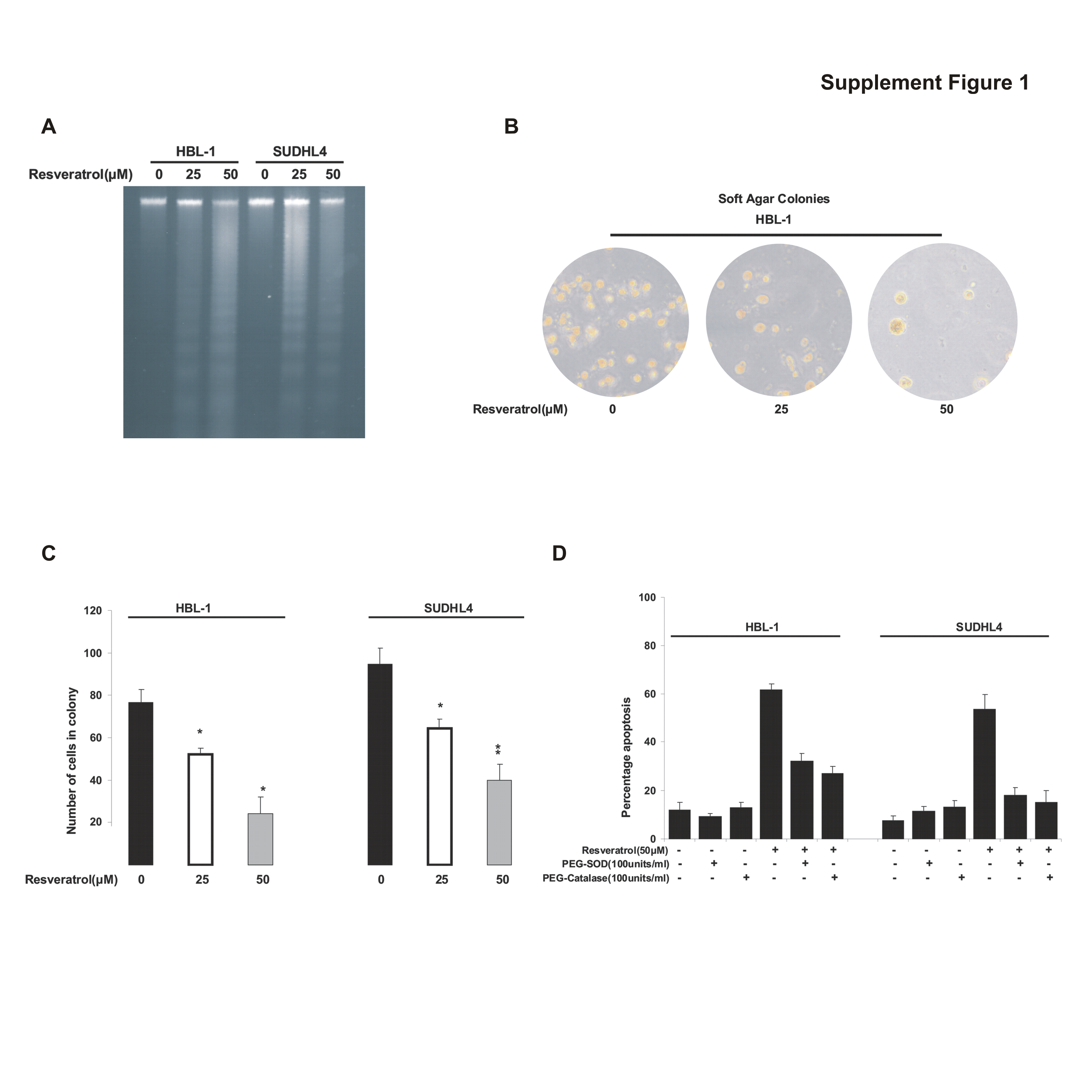

Supplement: Figure S1 — Anticancer effects of Resveratrol on DLBCL cells. (A) HBL-1 and SUDHL4 cells were treated with 25 and 50 µM Resveratrol for 24 hours and DNA was extracted and separated by electrophoresis on 1.5% agarose gel. (B) Clonogenic assays were performed as described in Materials and Methods. BC1 cells were treated with 25 and 50 µM Resveratrol for 24 hours. Subsequently, cells were plated in Soft agar plates for 4 weeks. Cells were stained and manually counted. (C) The bar graph displays the mean ± standard deviation of three independent experiments. * denotes statistically significant students ttest (p<0.05). (D) HBL-1 and SUDHL4 cells were treated with 50 µM Resveratrol for 24 hours in the presence or absence of pretreatment with PEG-catalase and PEG-superoxide dismutase for 2 hours. Following treatment, cells were stained with fluorescen-conjugated annexin V/PI and cells were analyzed by flow cytometry. Bar graph denotes a mean of three independent experiments. (TIF) [file pone.0024703.s001.tif]

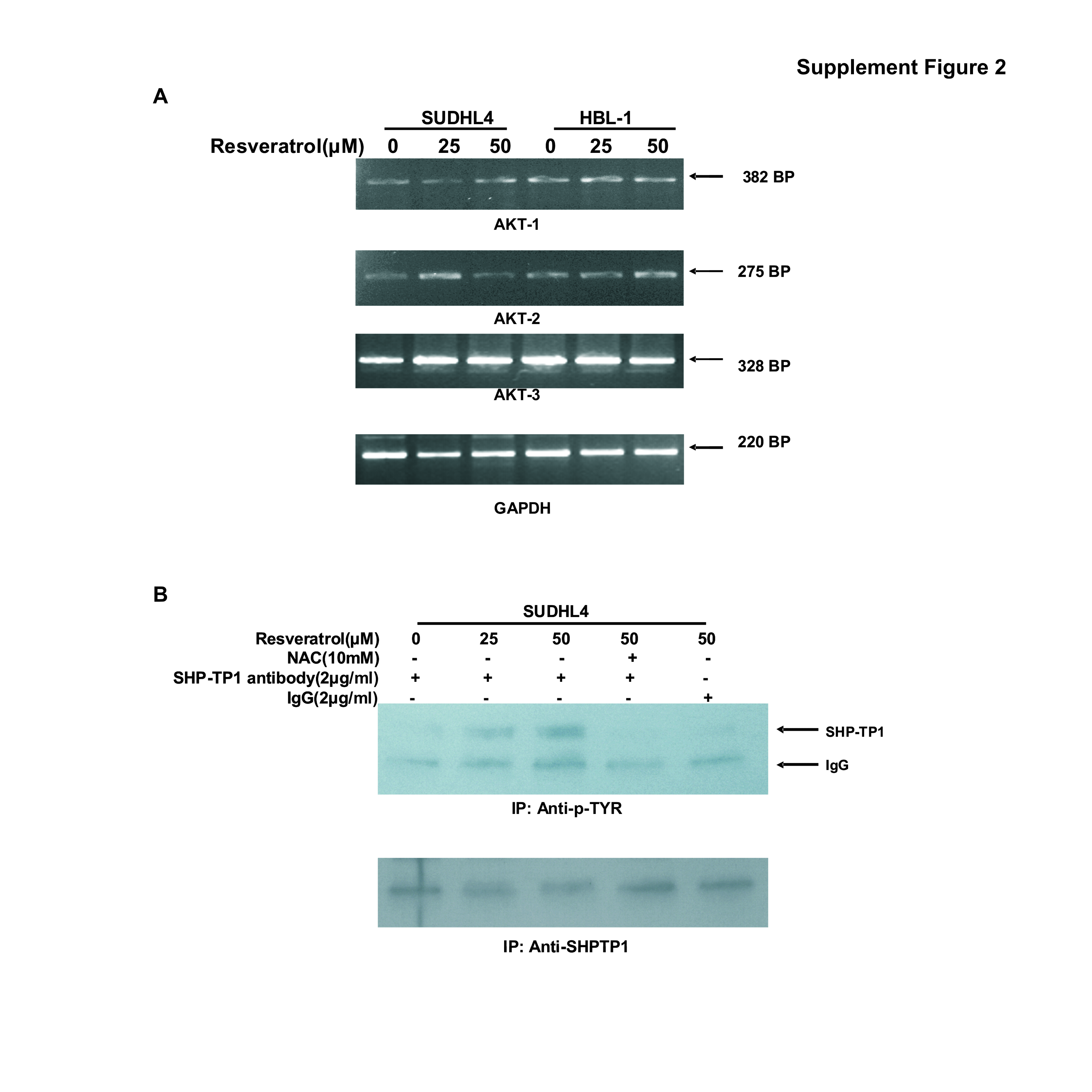

Supplement: Figure S2 — Effect of Resveratrol on AKT expression and SHP-TP1 activivity. (A) 5x10^6^ cells were treated with and without indicated doses of Resveratrol for 24 hours. RNA was isolated, and reverse transcribed as described in material and methods. Block RT-PCR for AKT1, AKT2 and AKT3 were performed for 35 cycles at 55°C. GAPDH was used as an internal control. (B) SUDHL4 cells were treated with either 25 and 50 µM Resveratrol or NAC and Resveratrol for 24 hours and cells were immuno-precipitated with SHP-TP1 antibody. Proteins were separated on SDS-Page and immuno-blotted with p-Tyrosine antibody and SHP-TP1 antibody. (TIF) [file pone.0024703.s002.tif]

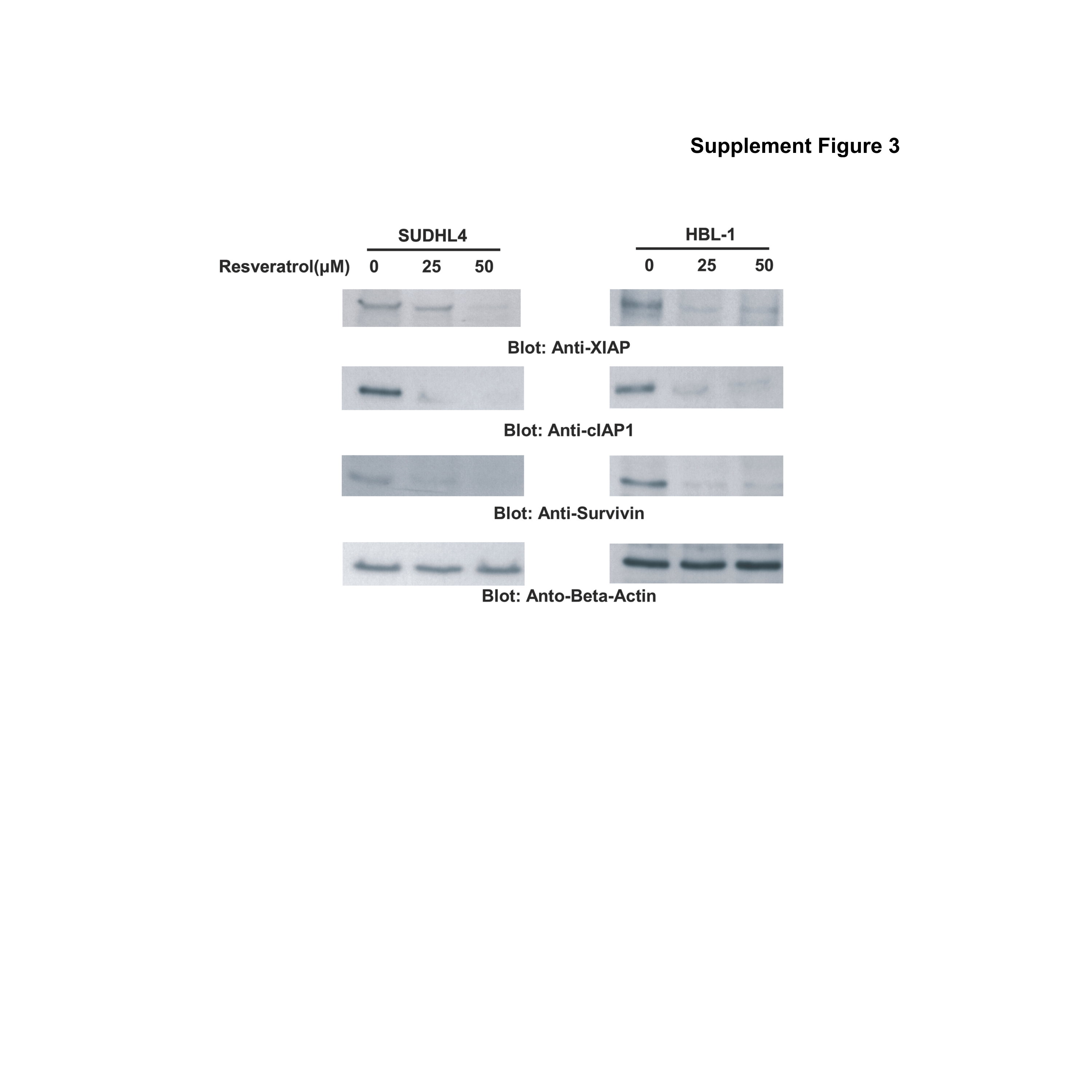

Supplement: Figure S3 — Resveratrol down-regulates expression of IAPs in DLBCL. HBL-1 and SUDHL4 cells were treated with 25 and 50 µM Resveratrol for 24 hours. Following incubation, cells were harvested and proteins were isolated that were separated on SDS-Page and immunoblotted with antibodies against XIAP, cIAP1, Survivin and beta-actin as indicated. (TIF) [file pone.0024703.s003.tif]
